# Houttuynia Essential Oil and its Self-Microemulsion Preparation Protect Against LPS-Induced Murine Mastitis by Restoring the Blood–Milk Barrier and Inhibiting Inflammation

**DOI:** 10.3389/fimmu.2022.842189

**Published:** 2022-02-18

**Authors:** Yuanyuan Liu, Yu Jiang, Yanfang Yang, Hongliang Wang, Jun Ye, Dongdong Liu, Yanmin Chen, Chunfang Lian, Renyun Wang, Yue Gao, Yingying Meng, Lili Gao, Yuling Liu

**Affiliations:** ^1^ State Key Laboratory of Bioactive Substance and Function of Natural Medicines, Institute of Materia Medica, Chinese Academy of Medical Sciences & Peking Union Medical College, Beijing, China; ^2^ Beijing Key laboratory of Drug Delivery Technology and Novel Formulation, Institute of Materia Medica, Chinese Academy of Medical Sciences & Peking Union Medical College, Beijing, China

**Keywords:** mastitis, blood–milk barrier, iNOS, ERK1/2, MAPK, houttuynia essential oil

## Abstract

Mastitis is a common inflammatory disease caused by bacterial infection to the mammary gland that impacts human and animal health and causes economic losses. Houttuynia essential oil (HEO), extracted from *Houttuynia cordata* Thunb, exhibits excellent antibacterial and anti-inflammatory properties. The aim of the study was to investigate the effects of HEO and a self-microemulsion preparation of HEO (SME-HEO) on inflammation and the blood–milk barrier (BMB) in lipopolysaccharide-induced murine mastitis. HEO and SME-HEO significantly downregulated pro-inflammatory factors TNF-α and IL-1β, upregulated anti-inflammatory factor IL-10, inhibited MPO expression, and alleviated histopathological injury in murine mammary gland tissues. Additionally, HEO and SME-HEO protected the integrity of the BMB by upregulating the expression of junction proteins ZO-1, claudin-1, claudin-3, and occludin. The anti-inflammatory effect of HEO against murine mastitis was mediated by blocking the MAPK signaling pathway and expression of iNOS. By inhibiting the release of inflammatory factors and protecting the integrity of the BMB, HEO may provide a novel treatment for mastitis.

## Introduction

Mastitis, which is mainly caused by pathogenic infection, can result in impaired mammary function and bacteriological changes in milk (1). In humans, mastitis mostly occurs in lactating women, seriously affecting their quality of life, breastfeeding capability, and newborn health, and also posing a burden on the healthcare system (2, 3). In animals, mastitis mostly occurs in dairy cattle, with an incidence rate of up to 33%. Mastitis in a dairy herd can severely impact the yield and quality of milk production, causing substantial economic losses each year and posing a human health hazard (4, 5).

Currently, antibiotics such as penicillin or cephalosporin are often used to treat mastitis in clinical practice, although glucocorticoid therapy is used in severe cases (6, 7). Antibiotic treatment not only fails to effectively inhibit the inflammatory response, but it also promotes the generation of drug-resistant bacteria, while hormone therapy may cause systemic toxic side effects. Moreover, drugs can be secreted in milk, resulting in economic loss due to discarded milk and posing a human health threat from exposure to drug residues. Realizing “anti-hormone and anti-antibiotic therapy” in mastitis has become a clinical problem of global concern that urgently needs to be solved.

*Houttuynia cordata* Thunb, a heat-clearing and detoxifying traditional Chinese medicine (TCM), has been widely used to treat ailments of the respiratory and digestive tracts and infectious diseases (8–10), demonstrating potent antibacterial, antiviral, and anti-inflammatory properties (9, 11, 12). Known as a natural antibiotic, houttuynia essential oil (HEO) is the main active constituent of *H. cordata* Thunb, of which houttuynin (Hou) has been identified as the main active component. However, because of its containing β-aldehyde-ketone structure, Hou is prone to degradation and polymerization (13), significantly reducing the efficacy and safety of HEO and hindering the clinical use of related preparations (14–16). To address these concerns, we hypothesized that a self-microemulsion of HEO (SME-HEO) might effectively improve the stability of HEO and exert a therapeutic effect against mastitis.

The blood–milk barrier (BMB) plays an important role in mammary gland function, which is adversely affected if the integrity of the BMB is compromised (17, 18). Studies have shown that lipopolysaccharide (LPS)-induced mastitis activates the MAPK and NF-κB signaling pathways (19–22), resulting in the destruction of mammary tissue structure and damage to BMB integrity (23). Inhibiting the release of inflammatory factors in mammary gland tissue and protecting the integrity of the BMB may play an important role in the treatment of mastitis. Therefore, the aim of this study was to investigate the effects of HEO and SME-HEO on the inflammatory response and BMB in a LPS-induced murine mastitis model.

## Materials and Methods

### Chemicals and Reagents

*H. cordata* Thunb was provided by Yichang Jiahao Ecological Agriculture Development Co., Ltd. (Wuhan, China). Labrasol and Transcutol HP were obtained from Gattefosse (Saint-Priest Cedex, France). LPS was obtained from Sigma Chemical Co. (St. Louis, MO, USA). Dexamethasone sodium phosphate (DEX) was obtained from Aladdin Biotech Co. Ltd. (Shanghai, China). The multifactor detection kit was purchased from Biolegend (San Diego, CA, USA). TRNzol total RNA extraction reagent was provided by Tiagen Biotech Co. Ltd. (Beijing, China). The primary antibodies claudin-3, claudin-1, occludin, and ZO-1 were obtained from Abcam (Cambridge, UK), while P65, p-P65, ERK1/2, p-ERK1/2, P38, p-P38, iNOS, COX2 and *β*-actin were purchased from Cell Signaling Technology (Danvers, MA, USA). The 10× electrophoretic buffer solution was purchased from Beijing Pleile Gene Technology Co., Ltd. (Beijing, China). PBS was purchased from Thermo Fisher Scientific (Waltham, MA, USA).

### Preparation of HEO

HEO was prepared from *H. cordata* Thunb as previously described 24).

### Preparation and Characterization of SME-HEO

HEO, Tween-80, Labrasol, and Transcutol HP were mixed together, vortexed, and then centrifuged at 12,000 rpm for 10 min to obtain SME-HEO.

To observe the microstructure of SME-HEO, an aliquot of SME-HEO was diluted 100× with distilled water at 37°C, and then dropped onto the surface of a 200 copper wire mesh. The excess liquid was absorbed with filter paper, the emulsion was negatively dyed with 2% phosphotungstic acid solution for 2 min, and then allowed to dry naturally. The microstructure of SME-HEO was observed *via* transmission electron microscopy.

To characterize the properties of the microemulsion, 100 μl SME-HEO was slowly added to 10 ml distilled water at 37°C with a stirring rate of 100 rpm. Complete emulsification was observed within 1 min. The average particle size, polydispersity coefficient (PDI), and zeta potential of the microemulsion was determined. Additionally, SME-HEO particle size was investigated at dilutions of 50, 100, 200, 400, and 800× in distilled water to evaluate the dilution stability of the microemulsion. Finally, the stability SME-HEO and HEO was investigated at 4 and 25°C for 15 days.

### Animals

Pregnant BALB/c mice (7–8 weeks old) were provided by SPF Biotech Co., Ltd. (Beijing, China). All animals were housed separately and provided free access to food and water. The animal experiments were performed in accordance with the Regulations on the Use of Experimental Animals by the Beijing Laboratory Animal Management Committee and were approved by the Laboratory Animal Welfare Ethics Committee (authorization number: 20210003YZA-3R) and Sino Animal Technology Development Co., Ltd. (Beijing, China).

### Animal Experimental Design

Lactating mice (3–4 days after birth) were randomly divided into nine groups (n = 6) (**Table 1**). Except for the positive group (drug administered on day 7), mice in the other groups were administered drug treatments by internal gavage once daily for 7 consecutive days, as shown in **Table 1**. One hour after the final drug administration, mice in the blank group were administered normal saline in the fourth pair of mammary glands by local intraperitoneal injection, while mice the other groups were injected with 50 μl LPS (0.2 mg/ml). One day after LPS injection, mice were sacrificed by cervical dislocation, and mammary gland tissues were observed and collected.

**Table 1:** Experimental groups and drug administration.

Group	Lable	Dose (mg/kg)	Form of administration
Blank	NT	/	Saline (i.g.)^b^
model	LPS	/	Saline (i.g.)^b^
positive	DEX	10	DEX (i.p.)^c^
HEO	HEO-L	100	HEO (i.g.)^b^
	HEO-M	200	
	HEO-H	300	
SME-HEO	SME-L	100^a^	SME-HEO (i.g.)^b^
	SME-M	200^a^	
	SME-H	300^a^	

a SME-HEO groups are described according to HEO dose.

b i.g., internal gavage.

c i.p., intraperitoneal injection.

### Histological Assessment

Mammary gland tissue was immersed in 4% formaldehyde solution for approximately 24 h, dehydrated using an ethanol gradient, embedded in paraffin, and sectioned. After dewaxing, hydration, hematoxylin staining, and eosin staining (H&E staining), tissue sections were observed under a light microscope to observe the degree of neutrophil infiltration in the acini of the mammary glands and the matrix density between acini.

Five visual fields were observed from each paraffin section, and damage was scored using the following criteria: 0, no abnormalities; 1, mild injury; 2, moderate injury; 3, severe injury; and 4, extensive severe damage.

### ELISA

Mammary gland tissue was ground with PBS in a 1:6 ratio, centrifuged twice for 30 min at 12,000 rpm, and the supernatant was collected. The inflammatory factors IL-23, TNF-α, IL-1α, IL-1β, IL-6, IL-17A, IFN-γ, MCP-1, IL-10, IL-12P70, IFN-β, GM-CSF, and IL-27 were detected using a 13-factor detection kit, according to the manufacturer’s instructions.

### Quantitative Reverse Transcription Polymerase Chain Reaction (qRT-PCR)

Total RNA was isolated from mammary gland tissue using TRNzol reagent, and amplification reactions were performed to detect levels of TNF-α and IL-1β. The primer sequences are listed in **Table 2**.

**Table 2:** Primer sequences for TNF-α, IL-1β and β-actin.

Item	Primer	Sequence (5' to 3')	Length (bp)
*TNF-a*	Sense	ACGGCATGGATCTCAAAGAC	116
*TNF-a*	Anti-sense	GTGGGTGAGGAGCACGTAGT	
*IL-1β*	Sense	TGCCACCTTTTGACAGTGATGA	135
*IL-1β*	Anti-sense	TGTGCTGCTGCGAGATTTGA	
*β-actin*	Sense	CGTTGACATCCGTAAAGACCTC	159
*β-actin*	Anti-sense	ACAGAGTACTTGCGCTCAGGAG	

### Immunohistochemistry (IHC)

Immunohistochemical staining was used to evaluate the distribution and expression of myeloperoxidase (MPO) and tight junction proteins ZO-1, claudin-1, claudin-3, and occludin in mammary gland tissue.

Paraffin sections were treated with citric acid antigen repair buffer solution (pH 6.0), followed by bovine serum albumin (3%) for 30 min at room temperature. The primary antibodies were added and sections were incubated overnight at 4°C. After washing the sections 3× with PBS (5 min per wash), the corresponding secondary HRP-labeled antibodies were added, and the sections were incubated at room temperature for 50 min. After washing 3× with PBS (5 min per wash), the sections were stained with DAB, followed by hematoxylin. The sections were then dehydrated and sealed for microscopic examination, and the images were collected and analyzed. Positive staining was indicated by the presence of yellow-brown color while nuclei appeared blue. The positive area ratio of each image was calculated.

### FITC-Albumin Evaluation

Mammary gland tissue was immersed in FITC-albumin solution for 30 min without light and then placed in liquid nitrogen. The frozen tissue block was balanced in a frozen slicer for 30 min, fixed to the base with OCT (a cryo embedding agent), and 5-μm thick frozen sections were cut. Finally, DAPI was added to the frozen sections and they were sealed. The sections were observed under a fluorescence microscope and the images were collected for data analysis.

### Western Blotting

Mammary gland tissue was mixed with protein lysate and ground at 50 Hz for 5 min, followed by centrifugation at 12,000 rpm for 10 min. Subsequently, the supernatant was collected and the protein concentration was determined using a BCA kit. The proteins in each sample (total protein 60 μg) were separated using 10% sodium dodecyl sulfate-polyacrylamide gel electrophoresis (SDS-PAGE), and then transferred to a PVDF membrane. The membrane was blocked with milk for 1 h and then incubated with primary antibody overnight at 4°C. After washing with TBST, the membrane was incubated with secondary antibody for 1 h, followed by the addition of hypersensitive chemiluminescent substrate. Finally, the target proteins were examined by X-ray exposure in a dark room.

### Statistical Analysis

All data were analyzed using Graph Pad Prism version 7.0 software (La Jolla, CA, USA) and were expressed as mean ± SD of six independent experiments. One-way analysis of variance (ANOVA) was used to determine significant differences between groups. Statistical significance was set at *P <*0.05 or *P <*0.01.

## Results

### Characteristics of SME-HEO

**Figure 1:**
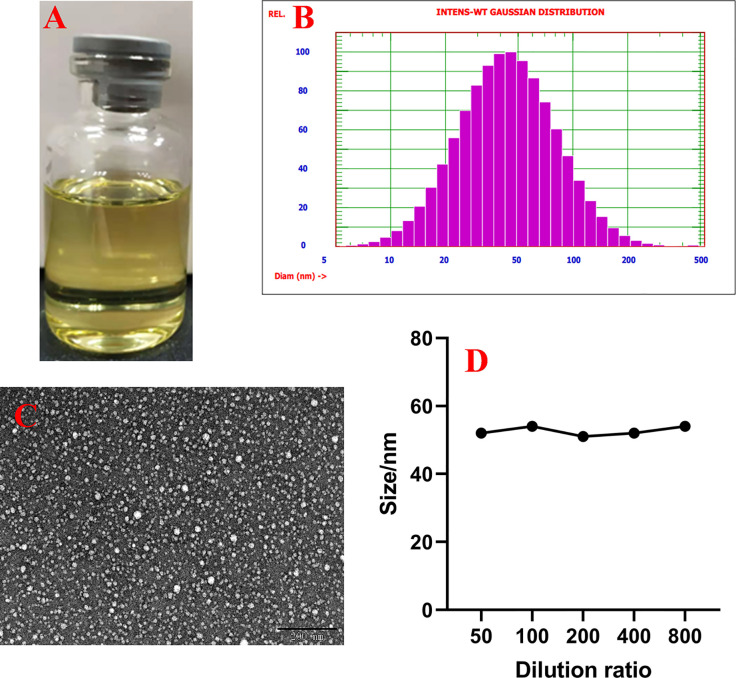
Characteristics of SME-HEO. **(A)** Appearance of SME-HEO. **(B)** Particle size and distribution of SME-HEO. **(C)** Microstructure of SME-HEO. **(D)** Relationship between particle size and dilution ratio of SME-HEO.

SME-HEO was transparent and homogeneous, as shown in **Figure 1A**. The microstructure of SME-HEO contained spherical, uniformly sized droplets without adhesion (**Figure 1B**). SME-HEO diluted in water yielded a transparent solution without turbidity. SME-HEO had an average particle size of 52.7 nm (**Figure 1C**), PDI of 0.384, and zeta potential of −27.71 mV. The particle size of SME-HEO diluted 50–800 times was stable at 50–55 nm (**Figure 1D**). The Hou content in HEO decreased to 26.04 and 21.46% of the original sample, while that in SME-HEO was 87.3 and 63.41% of the original sample, respectively, after 15 days at 4 and 5°C. The self-microemulsion delivery system significantly improves the stability of HEO.

### HEO and SME-HEO Alleviate Injury to Murine Mammary Gland Tissues

**Figure 2:**
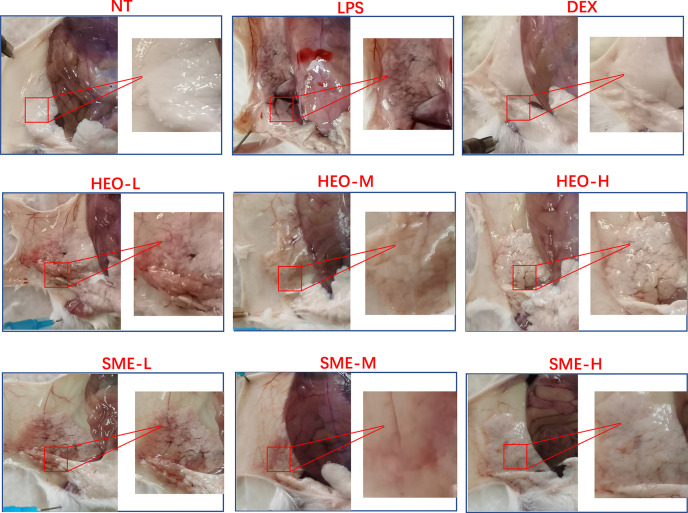
Morphology of mammary gland tissue in LPS-induced murine mastitis.

The appearance and pathological features of the mammary gland were evaluated after treatment with LPS, HEO, SME-HEO, and DEX. The morphology of the mammary gland is shown in **Figure 2**. The mammary glands in the NT group were milky white, intact, and exhibited no obvious abnormalities. Compared with the mammary glands in the NT group, those in the LPS group exhibited obvious swelling and hyperemia, suggesting that mastitis was successfully induced by LPS stimulation. Injury to the mammary gland was significantly improved in the DEX group. Redness and hyperemia in the mammary gland gradually decreased with increasing HEO and SME-HEO concentrations. The mammary glands were restored to healthy appearance in the high-dose HEO and SME-HEO groups.

**Figure 3:**
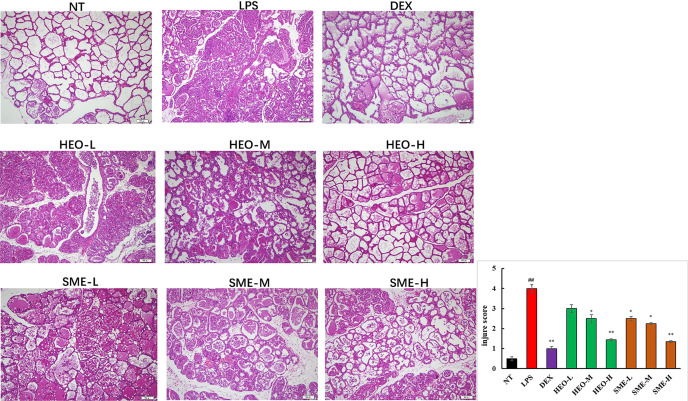
Representative images of pathological injury to mammary gland tissue in LPS-induced murine mastitis. Mammary gland tissues from each experimental group (n = 6) were obtained 24 h after LPS administration, sectioned, and stained with H&E (magnification ×100). (^##^ *P* < 0.01 compared to NT group, *P < 0.05, **P < 0.01 compared to model group).

The pathological features of mammary gland tissue were revealed through H&E staining (**Figure 3**). Compared with mammary gland tissue in the NT group, the LPS group exhibited severely damaged structures with proliferating glandular epithelial cells. However, these inflammatory symptoms were significantly alleviated in the HEO and SME-HEO groups in a dose-dependent manner. The anti-inflammatory effects exhibited in the SME-HEO groups were superior to those exhibited in the HEO groups at comparative low and medium doses.

### HEO and SME-HEO Downregulate Pro-Inflammatory Factors and Upregulate Anti-Inflammatory Factors

**Figure 4:**
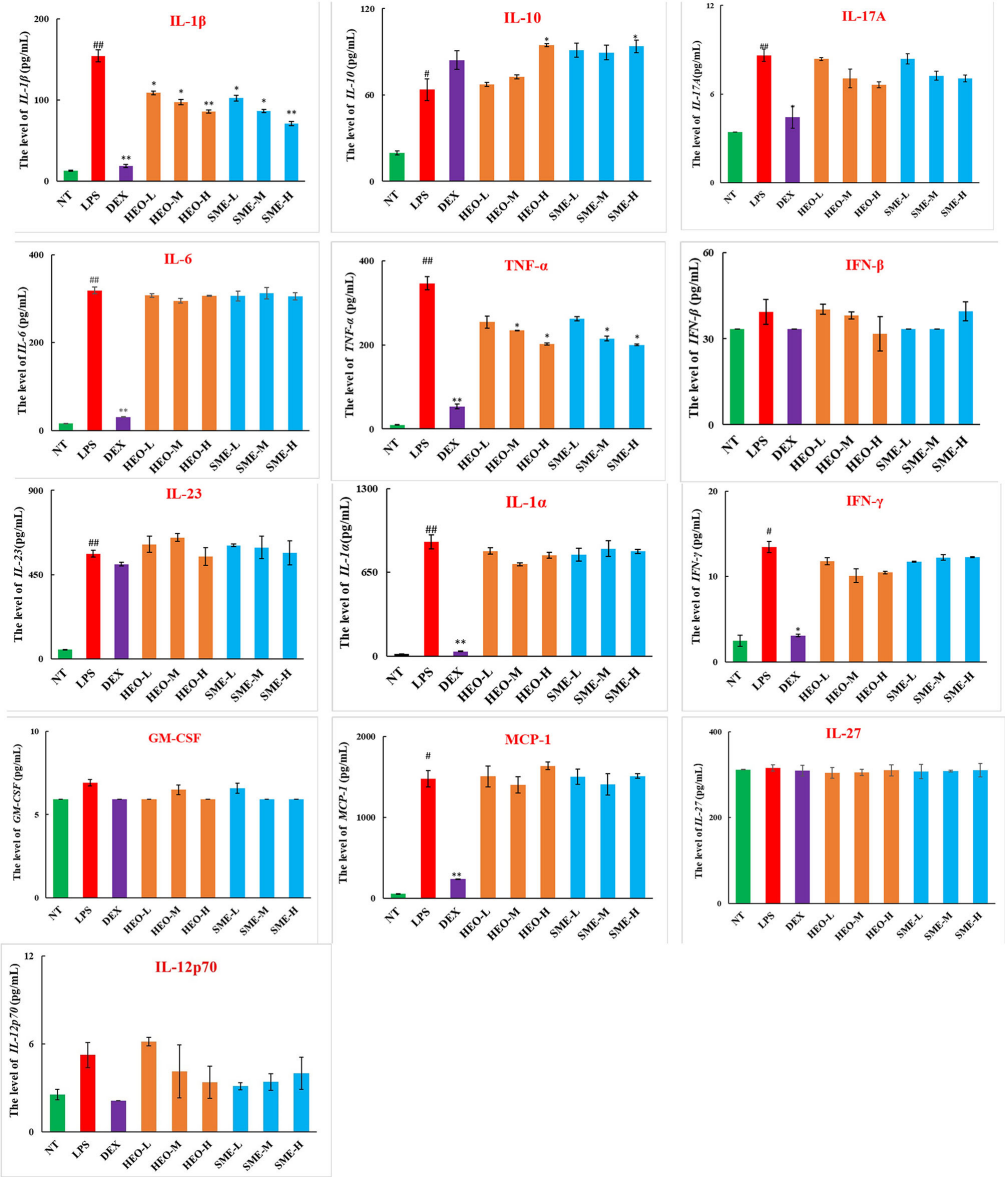
Expression of 13 inflammatory factors in LPS-induced murine mastitis. Mean ± SD, n = 6. ^#^ *P* < 0.05, ^##^ *P* < 0.01 compared to NT group, *P < 0.05, **P < 0.01 compared to model group.

As shown in **Figure 4**, nine pro-inflammatory cytokines (IL-23, TNF-α, IL-1α, IL-1β, IL-6, IL-17A, IFN-γ, MCP-1, and IL-10) were upregulated in the LPS group. However, TNF-α and IL-1β were significantly downregulated in the HEO and SME-HEO groups, and IL-10 was upregulated in the high-dose HEO and SME-HEO groups. Additionally, IL-1β expression was inhibited more in the SME-HEO groups than in the HEO groups at comparative doses. Meanwhile, mRNA expression of inflammatory factors TNF-α and IL-1β was consistent with the expression of the pro-inflammatory factors. That is, inhibition of TNF-α and IL-1β expression gradually increased with increasing HEO and SME-HEO concentrations (**Table 3**). SME-HEO tended to induce a greater inhibitory effect than HEO, but the results did not differ significantly.

**Table 3:** Upstream mRNA expression of TNF-α and IL-1β.

	Relative mRNA expression	
	TNF-α	IL-1β
NT	0.261 ± 0.019	0.209 ± 0.039
LPS	1.533 ± 0.424^##^	1.280 ± 0.165^##^
DEX	0.297 ± 0.134^**^	0.127 ± 0.016^**^
HEO-L	1.053 ± 0.479	0.904 ± 0.040^*^
HEO-M	0.935 ± 0.134^*^	0.782 ± 0.193^*^
HEO-H	0.754 ± 0.071^*^	0.457 ± 0.002^**^
SME-L	1.082 ± 0.131	0.813 ± 0.297^*^
SME-M	0.565 ± 0.285^*^	0.638 ± 0.297^*^
SME-H	0.648 ± 0.306^*^	0.357 ± 0.480^**^

Mean ± SD, n = 6, ^##^P compared to NT group, *P <0.05, **P <0.01 compared to model group.

### HEO and SME-HEO Inhibit MPO Expression

**Figure 5:**
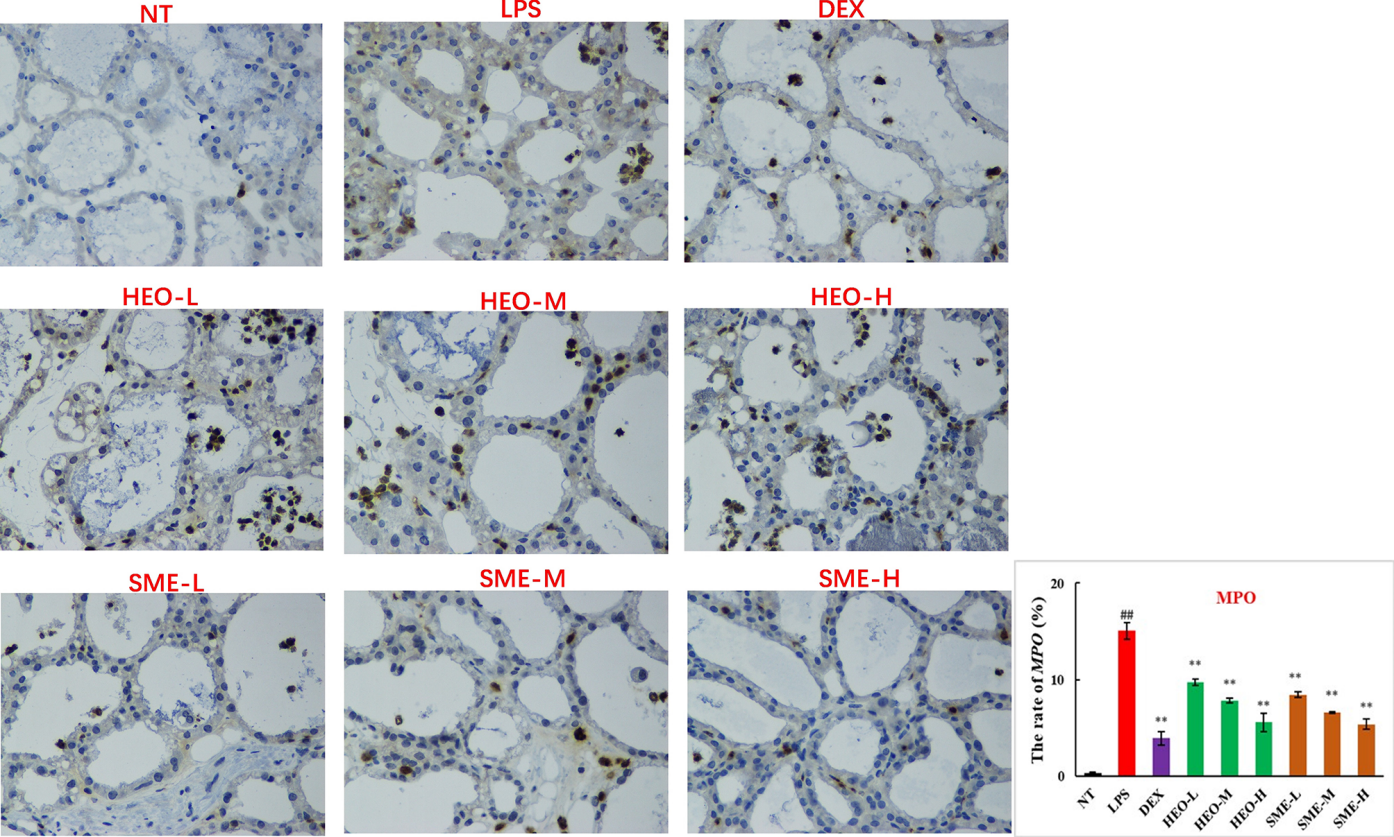
Distribution and expression of MPO in LPS-induced murine mastitis, indicated by brown or yellow colors. Mean ± SD, n = 6. ^##^ *P <*0.01, compared to blank group; **P <0.01 compared to model group.

MPO, a marker of neutrophil function and activity, can participate in the production of oxygen free radicals, thereby causing tissue damage and exacerbating the inflammatory response (25, 26). MPO distribution in mammary gland tissue was observed *via* IHC. LPS stimulation significantly increased the expression of MPO in mammary gland tissue (**Figure 5**). However, MPO expression decreased gradually with increasing concentrations of HEO and SME-HEO. SME-HEO inhibited MPO expression more than HEO at comparative doses.

### HEO and SME-HEO Protect the Blood–Milk Barrier

The BMB is mainly composed of endothelial cells, basement membranes, epithelial cells, and tight junction proteins. The BMB prevents the invasion of pathogenic microorganisms, leakage of milk components, and limits the random exchange between milk and blood components (27, 28).

**Figure 6:**
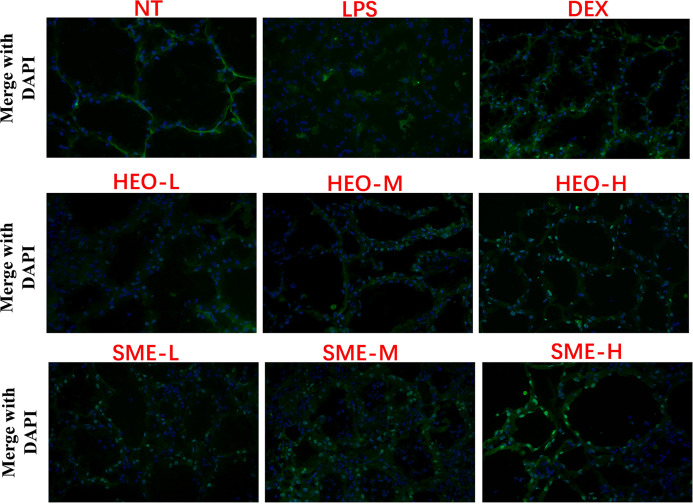
Effects of HEO and SME-HEO on the blood–milk barrier in LPS-induced murine mastitis. Green and blue colors indicate FITC-albumin and nuclei, respectively.

Fluorescence staining was used to observe the distribution of FITC-albumin as a reflection of BMB integrity. The greater the infiltration of FITC-albumin, the more serious the damage to the BMB. As shown in **Figure 6**, the BMB in the NT group was intact without infiltration, while the cinar profile in the LPS group was blurred. Damage to BMB integrity was indicated by FITC-albumin distributed in the interacinar matrix diffusing into the acinar lumen. However, FITC-albumin infiltration gradually improved with HEO and SME-HEO treatment in a dose-dependent manner. These results indicated that HEO and SME-HEO exerted protective effects against LPS-induced damage to the BMB.

### HEO and SME-HEO Upregulate Expression of Tight Junction Proteins ZO-1, Claudin-1, Claudin-3, and Occludin

**Figure 7:**
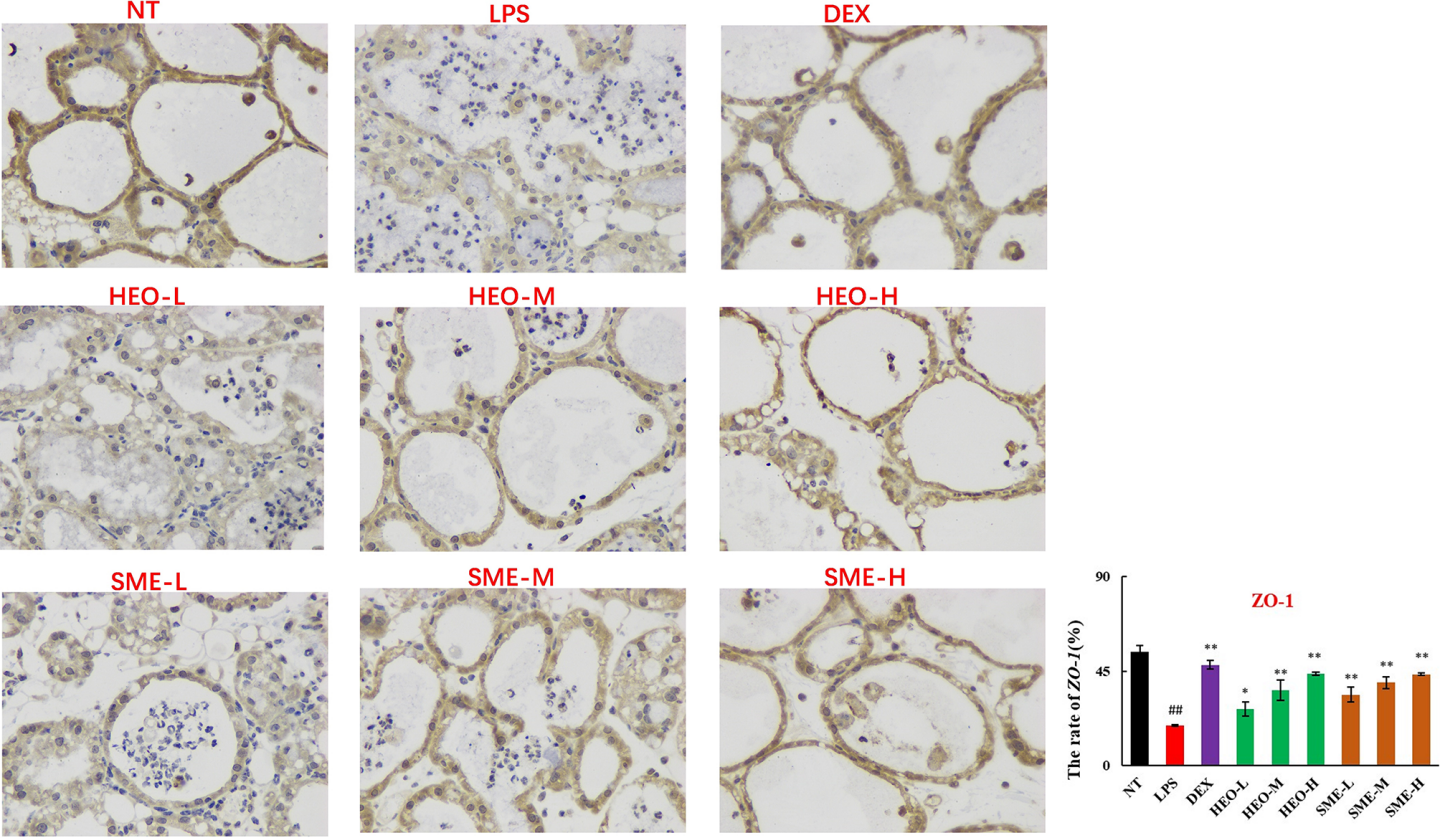
Distribution and expression of connexin ZO-1 in LPS-induced murine mastitis, indicated by brown or yellow colors. Mean ± SD, n = 6. ^##^ *P <*0.01 compared to blank group; *P <0.05, **P <0.01 compared to model group.

**Figure 8:**
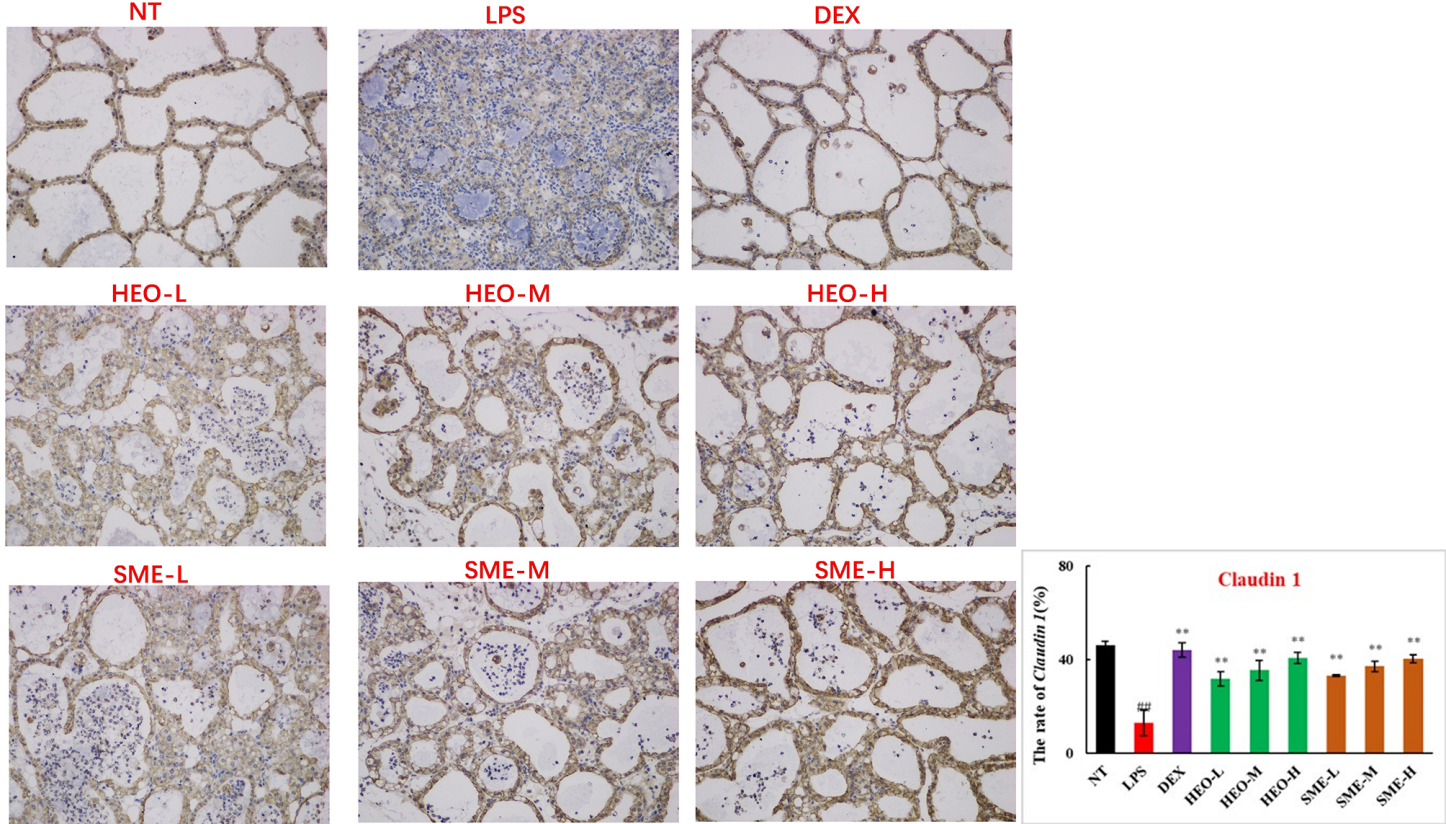
Distribution and expression of connexin Claudin 1 in LPS-induced murine mastitis, indicated by brown or yellow colors. Mean ± SD, n = 6. ^##^ *P <*0.01 compared to blank group; **P <0.01 compared to model group.

**Figure 9:**
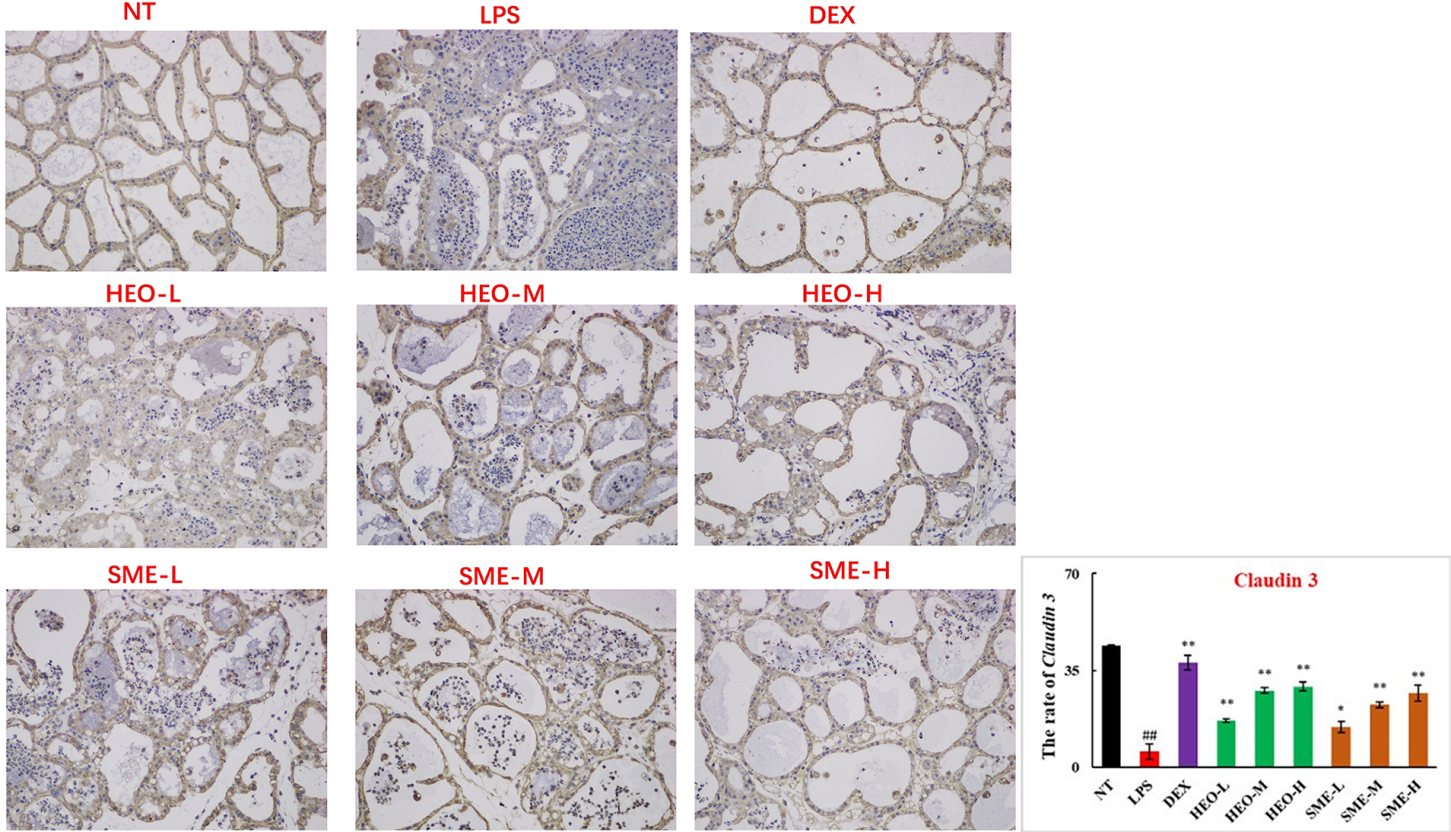
Distribution and expression of connexin Claudin 3 in LPS-induced murine mastitis, indicated by brown or yellow colors. Mean ± SD, n= 6. ^##^ *P <*0.01 compared to blank group; *P <0.05, **P <0.01 compared to model group.

**Figure 10:**
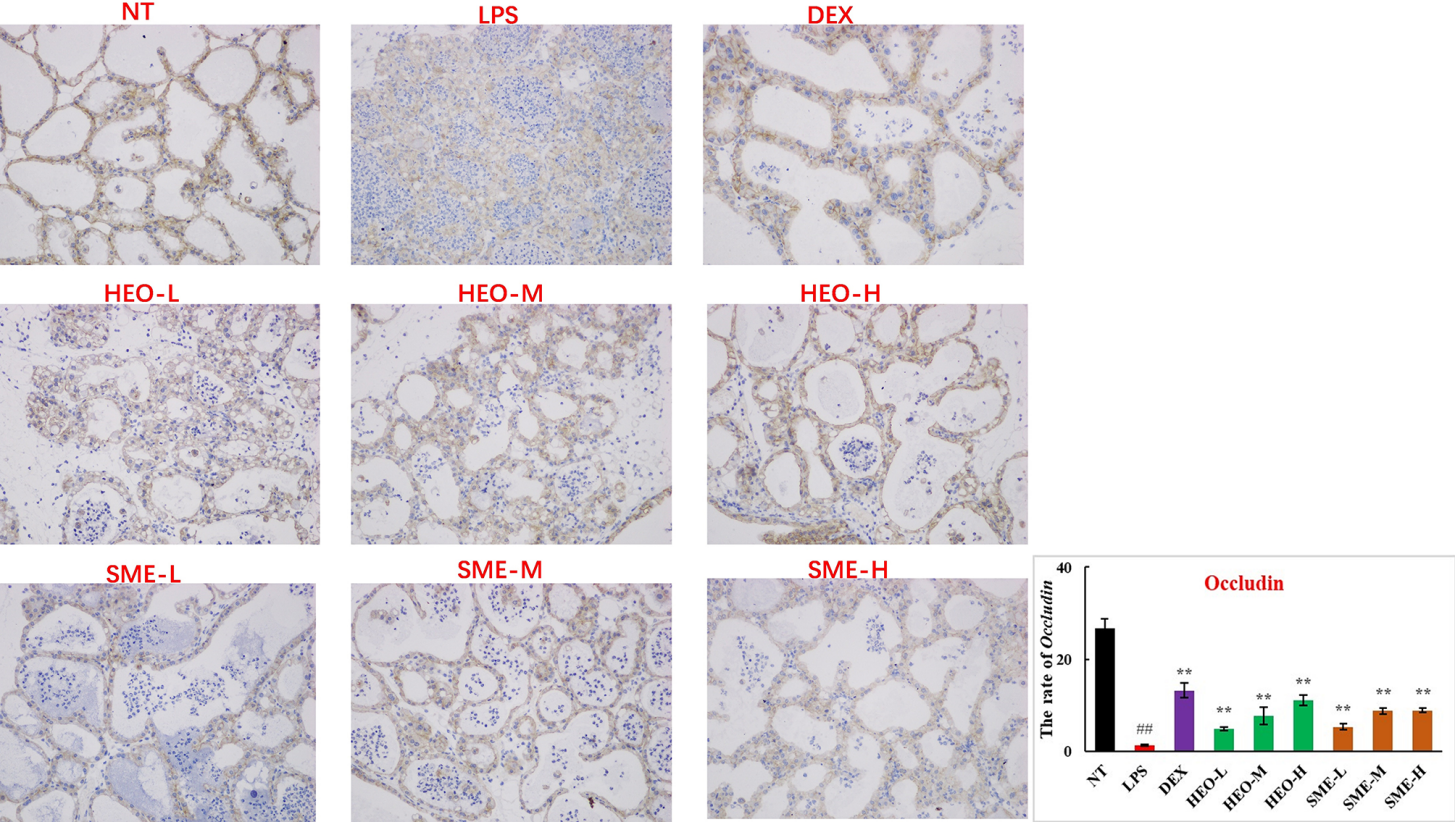
Distribution and expression of connexin Occludin in LPS-induced murine mastitis, indicated by brown or yellow colors. Mean ± SD, n = 6. ^##^ *P <*0.01 compared to blank group; **P <0.01 compared to model group.

Connexin proteins such as ZO-1, claudin-1, claudin-3, and occludin are important components of the tightly connected structure of the BMB (29, 30). The mechanism by which HEO and SME-HEO protect the BMB was investigated through the distribution and expression of connexins in mammary gland tissue *via* IHC. The expression of ZO-1 protein was severely reduced in the LPS group (**Figure 7**). However, ZO-1 expression increased significantly with increasing doses of HEO and SME-HEO. The expression of claudin-1 (**Figure 8**), claudin-3 (**Figure 9**), and occludin (**Figure 10**) demonstrated similar trends as that of ZO-1. The occludin protein content was approximately 28% less than that of the other three connexin proteins. These results suggested that HEO and SME-HEO protected the integrity of the BMB by increasing protein levels of ZO-1, claudin-1, claudin-3, and occludin in mammary gland tissue.

### HEO Inhibits Expression of the INOS and ERK1/2/MAPK Pathways

The expression of nitric oxide induction-type synthase (iNOS) and cyclooxygenase-2 (COX-2) is closely associated with inflammation. A large amount of NO induced by iNOS can mediate and promote the development of inflammatory diseases (31, 32). Moreover, COX-2 catalyzes the conversion of arachidonic acid into prostaglandins and participates in activation of the NF-κB pathway (33). To further explore the mechanism by which HEO alleviates mastitis, the expression of iNOS, COX-2, and NF-κB- and MAPK-related pathway proteins was investigated by western blotting in mammary gland tissue.

**Figure 11:**
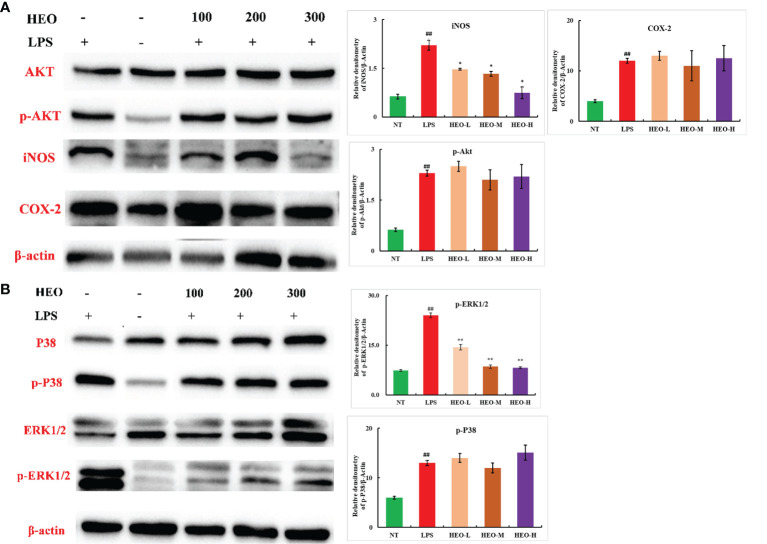
Effects of HEO on proteins associated with inflammatory pathways. **(A)** HEO inhibits iNOS expression. **(B)** HEO hinders ERK1/2 phosphorylation. Mean ± SD, n = 6. ^##^ *P <*0.01 compared to blank group; *P <0.05, **P <0.01 compared to model group.

The expression of p-Akt, p-ERK1/2, p-P38, iNOS, and COX-2 proteins in mammary gland tissue increased significantly after LPS stimulation (**Figure 11**). The expression of iNOS and p-ERK1/2 proteins was significantly inhibited by HEO in a dose-dependent manner, suggesting that HEO played an anti-inflammatory role by inhibiting iNOS expression, ERK1/2 phosphorylation, and blocking the MAPK signaling pathway.

## Discussion

Mastitis commonly occurs in lactating women and has a high rate of incidence in humans and dairy cattle. At present, antibiotics are often used to treat mastitis, resulting in the misuse of antibiotics, development of drug-resistant bacteria, and other problems that may pose harm to human society. TCMs contain a variety of active substances and nutrients that can enhance immune capacity, exerting antibacterial and anti-inflammatory effects with minimal side effects and without generating drug resistance. Therefore, TCMs have become the focus of research in mastitis treatment.

Although HEO exhibits potent antibacterial and anti-inflammatory properties, its application in mastitis treatment has not been systematically studied. Meanwhile, the problem of HEO instability needs to be solved to improve its efficacy. On this basis, LPS was used to stimulate inflammation in murine mammary tissue, and various detection techniques were used to confirm that HEO and SME-HEO reduced the mammary inflammatory response, protected the BMB, and alleviated the symptoms of mastitis. Further mechanistic studies revealed that HEO inhibited the expression of iNOS, phosphorylation of ERK1/2, and blocked the inflammatory MAPK signaling pathway.

Cytokines can stimulate significant upregulation of adhesion molecules in vascular endothelial cells and induce recruitment of neutrophils and macrophages to the infection site (34). In this study, 13 inflammatory factors in mammary gland tissue were detected by ELISA. The results suggested that HEO and SME-HEO treatment induced bidirectional regulation of inflammatory factors, by inhibiting the release of TNF-α and IL-1β, while upregulating the expression of IL-10. The mRNA levels of TNF-α and IL-1β detected by qRT-PCR were consistent with the ELISA results. These findings confirmed that HEO and SME-HEO inhibited the expression of TNF-α and IL-1β, reduced recruitment of neutrophils and macrophages to mammary lesions, and reduced damage to mammary tissue, thus exerting a protective effect against LPS-induced murine mastitis.

MPO is a member of the heme peroxidase superfamily, and its expression level and activity can reflect the state of neutrophil infiltration (21, 22). HEO and SME-HEO significantly reduced MPO expression in mammary gland tissue, and the efficacy of SME-HEO was better than that of HEO at corresponding doses. These results suggest that HEO and its self- microemulsion preparation may inhibit neutrophil aggregation in infected mammary tissue.

BMB has important physiological functions (27–30), and maintaining its integrity is conducive to alleviating the mammary inflammatory response (35). The results of this study suggested that HEO and its self-microemulsion preparation protected the integrity of the BMB by upregulating the expression of tight junction proteins ZO-1, claudin-1, claudin-3, and occludin.

ERK1/2, a MAPK signaling pathway, is extensively involved in regulation of cell proliferation and differentiation, and plays an important role in the propagation and treatment of microorganisms (36, 37). For example, the levels of p-ERK are significantly increased in patients with Epstein–Barr virus (EBV)-related gastric cancer (38). Additionally, blocking the ERK1/2 pathway significantly reduces the expression of EBV latent membrane protein 2A, suggesting that the ERK1/2 pathway is also associated with infection-induced tumors. iNOS, as an intermediate of the inflammatory response, is expressed in pathological states such as inflammation, hypoxia, and tumors, and catalyzes a large amount of NO production in a short period of time. NO plays an important role in substance metabolism, information transmission, and disease prevention, and is closely related to the inflammatory response. Thus, NO content can be used as an indicator of the degree of the inflammatory response (32, 39, 40). In the current study, HEO significantly reduced iNOS and p-ERK expression, indicating that HEO plays an anti-inflammatory role by blocking activation of the MAPK and other inflammatory signaling pathways. We speculate that HEO may have an effect on inflammatory diseases and tumors related to ERK pathway activation due to microbial infection.

In conclusion, the study findings support that HEO and SME-HEO exert therapeutic effects against LPS-induced murine mastitis, and that self-microemulsion of HEO demonstrated superior anti-inflammatory effects to bulk HEO to some extent. This study provides a novel strategy for the clinical treatment of mastitis without antibiotics or hormone therapy.

## Data Availability Statement

The original contributions presented in the study are included in the article/supplementary material. Further inquiries can be directed to the corresponding author.

## Ethics Statement

The animal study was reviewed and approved by the Laboratory Animal Welfare Ethics Committee approved by the Sino Animal (Beijing) Technology Development Co., Ltd.

## Author Contributions

YJ, HW, YC, CL, RW, DL, YG, and YuaY helped with the experiments. YF and YulL revised the contents of the article. All authors listed have made a substantial, direct, and intellectual contribution to the work and approved it for publication.

## Funding

We are grateful for the financial support from the National Megaproject for Innovative drugs (Nos. 2018ZX09711001-002-005 and 2018ZX09721003-009-001) of the Chinese government and the CAMS Innovation Fund for Medical Sciences (No. CIFMS-2019-12M-1-005).

## Conflict of Interest

The authors declare that the research was conducted in the absence of any commercial or financial relationships that could be construed as a potential conflict of interest.

## Publisher’s Note

All claims expressed in this article are solely those of the authors and do not necessarily represent those of their affiliated organizations, or those of the publisher, the editors and the reviewers. Any product that may be evaluated in this article, or claim that may be made by its manufacturer, is not guaranteed or endorsed by the publisher.
